# Association between body mass index and frailty for middle-aged and older adults in Japan: a cross-sectional study of the Osaka health disparity solution program

**DOI:** 10.1186/s12889-026-27331-2

**Published:** 2026-04-23

**Authors:** Tsukasa Yoshida, Erika Hikita, Daiki Watanabe, Takashi Nakagata, Yosuke Yamada, Naomi Sawada, Megumi Okabayashi, Hidekazu Shimada, Nobuo Nishi, Motohiko Miyachi

**Affiliations:** 1 Graduate School of Medicine, Tohoku University, Seiryomachi 2-1, Aoba-ku, Sendai-city, Miyagi 980-8575 Japan; 2 National Institute of Health and Nutrition, National Institutes of Biomedical Innovation, Health and Nutrition, Senriokashinmachi 3-17, Settsu-city, Osaka 566-002 Japan; 3 Japan International Cooperation Agency, Nibancho 5-25, Chiyoda-city, Tokyo 102-0084 Japan; 4 National Institute of Biomedical Innovation, National Institutes of Biomedical Innovation, Health and Nutrition, Saitoasagi 7-6-8, Ibaraki-city, Osaka 567-0085 Japan; 5 Graduate School of Biomedical Engineering, Tohoku University, Seiryomachi 2-1, Aoba-ku, Sendai-city, Miyagi 980-8575 Japan; 6 Department of Health and Welfare, Settsu City Local Government, Mishima 1-1-1, Settsu-city, Osaka 566-0022 Japan; 7 Department of Health and Medical, Osaka Prefectural Government, Otemae 2-1-22, Osaka-city, Osaka 540-8570 Japan; 8 Graduate School of Public Health, St. Luke’s International University, OMURA Susumu and Mieko Memorial St. Luke’s Center for Clinical Academia, Tsukiji 3-6-2, Chuo-city, Tokyo 104-0045 Japan; 9 Faculty of Sport Sciences, Waseda University, Mikajima 2-579-15, Tokorozawa-city, Saitama 359-1192 Japan

**Keywords:** Kihon Checklist (KCL), Frailty screening index (FSI) or simplified frailty index (SFI), Logistic regression, Restricted cubic spline analysis, Asia

## Abstract

**Background:**

Low and high body mass index (BMI) are reported to be associated with frailty in older adults. Since the optimal BMI associated with a low risk of mortality varies with age, the association between BMI and frailty might also differ by age. This study examined the association between BMI and frailty in middle-aged and older adults in Japan.

**Methods:**

We conducted face-to-face and mail surveys in Settsu city and mail survey in Hannan city in Osaka, Japan. The association between BMI and frailty was analyzed among 8,815 participants using mail surveys. Frailty was evaluated using two tools, the Kihon Checklist (KCL) and the Frailty Screening Index (FSI). BMI (kg/m^2^) was categorized into the < 18.5, ≥ 18.5–<20.0, ≥ 20–<22.5, ≥ 22.5–<25.0, ≥ 25.0–<27.5, and ≥ 27.5 kg/m^2^ groups. We analyzed the association between BMI and frailty using multivariable logistic regression, with BMI ≥ 22.5–<25.0 kg/m^2^ as the reference to calculate the odds ratios (OR) and 95% confidence intervals (95%CI). Restricted cubic spline analyses were also performed, with knots placed at the 5th, 50th (as reference), and 95th percentiles of BMI. We performed all analyses separately for the < 65 and ≥ 65 years age groups.

**Results:**

BMI < 18.5 (OR = 1.882, 95%CI: 1.263–2.805) was significantly associated with KCL-measured frailty in individuals aged < 65 years, and BMI < 18.5 (OR = 1.807, 95%CI: 1.291–2.531) and ≥ 27.5 (OR = 1.562, 95%CI: 1.156–2.111) were significantly associated with KCL-measured frailty in those aged ≥ 65 years. BMI ≥ 25.0–<27.5 (OR = 1.426, 95%CI: 1.055–1.927) and ≥ 27.5 (OR = 1.473, 95%CI: 1.093–1.985) were significantly associated with FSI-measured frailty in individuals aged < 65 years, and BMI ≥ 27.5 (OR = 1.988, 95%CI: 1.432–2.759) was significantly associated with frailty in those aged ≥ 65 years. The spline models showed U-shaped associations for KCL-measured frailty for both age groups, a positive linear association for FSI-measured frailty among those aged < 65 years, and an L-shaped association for FSI-measured frailty among those aged ≥ 65 years.

**Conclusion:**

The association between BMI and frailty differed by age. The collation of all the results of this study suggests that both low and high BMI are associated with frailty in middle-aged and older adults. Based on the results, it is speculated lifestyle habits that promote proximity to “normal weight” may help prevent frailty.

**Trial registration:**

UMIN000008105 (Registration date: May 29th 2019; Website: https://upload.umin.ac.jp/cgi-open-bin/ctr_e/ctr_view.cgi?recptno=R000042027).

**Supplementary Information:**

The online version contains supplementary material available at 10.1186/s12889-026-27331-2.

## Background

Body mass index (BMI) is a parameter that utilizes height and weight to evaluate thinness and obesity. The optimal BMI differs depending on race, and both low and high BMI values are associated with an elevated risk of mortality [1, 2]. Gaining insights into BMI can influence changes in individual behavior and population health policies.

Frailty increases vulnerability to stress [3] and increases mortality and the need for long-term care [4–6]. It is composed of multiple factors, viz. a decline in physiological physical function as well as the cognitive, emotional or psychosocial domains [3]. Although frailty is a syndrome that occurs more frequently in older people [7], studies from Western [8–14] and Asian countries, such as India [15] and Japan [16], have revealed that it can also occur in younger adults. Furthermore, analysis of the US NHANES data revealed that the incidence of frailty has increased across a wide range of age groups [17]. It is clear that the number of people with frailty will increase in the future, necessitating prevention at an early stage.

The vicious cycle of frailty includes sarcopenia as the key component, along with malnutrition, which is associated with sarcopenia [18]; therefore, a low BMI is likely to be a risk factor for frailty. Data from the UK Biobank comprising 500,000 volunteers aged 37–73 years reported that frailty assessed using the Fried phenotype criteria is associated with both low and high BMI [13]. However, the average BMI varies by race [19], and the UK data may not be applicable to all populations. Nishida et al. [20] analyzed the medical insurance receipt data of four million individuals aged ≥ 35 years in Japan and reported that frailty assessed by the Hospital Frailty Risk Score [21] was associated with both low and high BMI. However, the characteristics of the study using medical-insurance receipts differ from those of studies conducted using community-dwelling cohorts, as the latter utilized a medical facility-specific frailty index created from daily clinical data based on the International Classification of Diseases, 10th version, but does not encompass all the domains of frailty such as physical, psychological, and social functions. Kuan et al. [22] reported an increase BMI trajectory from overweight to obesity was associated with lower odds of the healthy aging defined as major chronic diseases, cognitive impairment, activities of daily living, and mental health, as an outcome, the group of BMI slightly increased by showing a trajectory from overweight to obesity had significantly lower odds of healthy aging. Although the definitions of frailty and healthy aging are different, the composition of healthy aging is similar to that of comprehensive frailty. The phenotypic and deficit-accumulation models are primarily used to measure frailty [18, 23], each of which emphasizes different aspects; therefore, the use of multiple frailty tools facilitates better evaluation [24]. Because frailty is reversible, screening is important for early interventions. Watanabe et al. [25] reported that low and high BMI were associated with frailty, which was assessed using the physical frailty-based modified Frailty Screening Index (FSI) (also called the Simplified Frailty Index; SFI) [26], and the comprehensive Kihon Checklist (KCL) for frailty [27]. However, the study cohort was limited to patients aged ≥ 65 years. A systematic review of 230 cohorts found that the optimal BMI associated with a low risk of mortality changes with age [28]; hence, it is unclear whether the association between BMI and frailty in older adults can be applied to middle-aged individuals. Furthermore, as the manifestations of frailty may differ between older and younger individuals [29], it is important to clarify the association between BMI and frailty in middle-aged individuals from a public health policy perspective.

The purpose of this study was to clarify the association between BMI and frailty using two frailty assessment tools in middle-aged individuals in Japan, with a focus on regional characteristics.

## Methods

### Participants and data

This cross-sectional study was conducted using face-to-face and mail surveys.

**Fig. 1:**
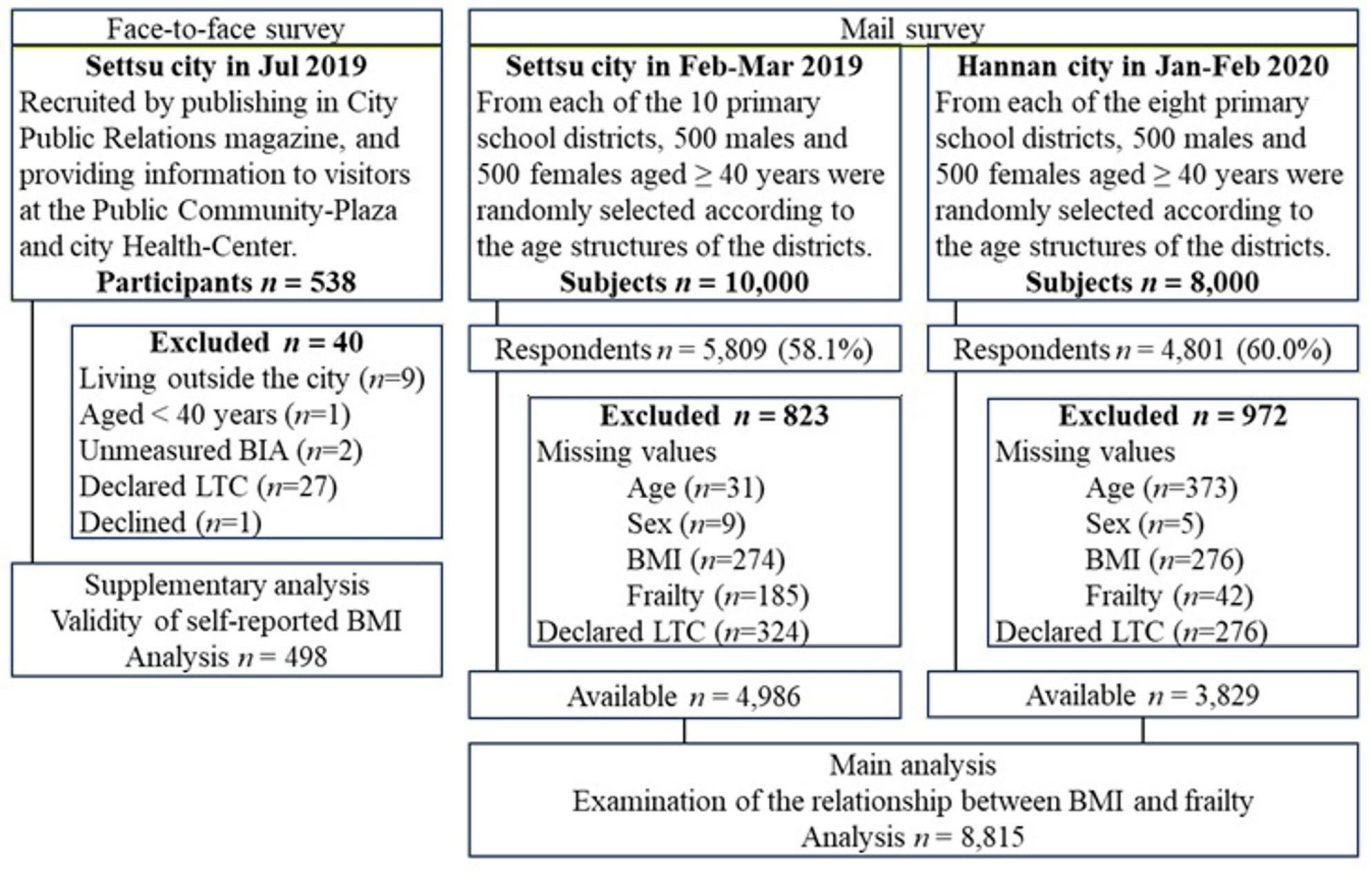
Flow chart of study enrollment. Declared LTC, Declared public certification of long-term care (including support needs); unmeasured BIA, missing weight data due to unmeasured bio-electrical impedance analysis due to the presence of an artificial cardiac pacemaker or limb injury

The face-to-face survey was conducted in July 2019 at the Public Community-Plaza in individuals aged ≥ 40 years residing in Settsu city, Osaka prefecture, Japan [30, 31]. The research information, including recruitment, was communicated to potential candidates via announcements in the Settsu City Public Relations magazine and dissemination of information to visitors to the Public Community-Plaza and a specific health checkup venue at the city Health-Center located next to the Public Community-Plaza. Participants provided consent after receiving an explanation about the study. They answered a questionnaire designed for this study that included the self-reported height, weight, KCL, and FSI. Thereafter, we objectively measured the participants’ height using general height meter, and weight and body composition using the BIA body composition analyzer (Tanita MC780A, Tokyo, Japan) [30, 31]. Initially, 538 individuals participated in the study. We excluded individuals aged < 40 years, who lived outside the city, who were unable to undergo measurement of body composition using the BIA analyzer due to cardiac pacemaker implantation or injury to the limbs, those who declared long-term care/support need on the questionnaire, and those who withdrew consent. Ultimately, 498 participants were included in the analysis (Fig. 1).

The mail survey included participants aged ≥ 40 years who resided in Setsu city from February to March 2019, and in Hannan city from January to February 2020; both cities are located in Osaka prefecture, Japan. The details of these surveys have been reported previously [16, 30, 32]. City-office workers selected 500 men and 500 women from each primary school district according to the age distribution of the residents. There were 10 and 8 school districts in Settsu and Hannan, respectively, and 18,000 people were selected. An anonymous questionnaire designed for this study containing socio-demographic information, health status, lifestyle habits, eating habits, awareness of words related to health, the KCL, and the FSI was mailed to selected participants, which yielded response rates of 58.1% and 60.0% in Settsu and Hannan, respectively [16, 30, 32]. After excluding individuals with missing age, sex, BMI, those who were unable to assess frailty, and those who declared long-term care/support need on the questionnaire, 8,815 individuals were included in the analysis (Fig. 1).

### BMI

BMI was calculated by dividing the body weight (kg) by the square of the height (m). In the face-to-face survey, BMI was calculated using the self-reported and objectively measured height and weight values, respectively. In the mail survey, the self-reported height and weight were used to calculate the BMI.

### Assessment of frailty

Frailty is mainly categorized into phenotype model which commonly called physical frailty [33], and the deficit accumulation model which commonly called comprehensive frailty [23]. The two main definitions are considered complementary [34]. Frailty was assessed using the KCL [27] and the FSI [26]. The KCL is a 25-item, self-report questionnaire with seven subdomains covering instrumental activities of daily living decline, locomotor dysfunction, malnutrition, oral dysfunction, social isolation, cognitive decline, and depression. Previous study described KCL as one of the most accurate aging indicators [35]. A diagnosis of frailty was made if the KCL score was ≥ 7, which has been validated by Fried’s CHS criteria and mortality [6, 36]. The FSI is a 5-item self-report questionnaire and scores ≥ 3 indicated frailty, which was validated by the public long-term care certificate [26]. FSI was developed based on Fried’s CHS criteria, a self-reported questionnaire assessment that can be administered by mail [26]. Although Fried’s CHS criteria have been widely used, a drawback exists as some metrics, such as grip strength and gait speed, require actual measurement.

### Statistical analysis

The characteristics of participants who underwent the face-to-face and mail surveys were classified by age (< 65 and ≥ 65 years), BMI (< 18.5, ≥ 18.5–<20.0, ≥ 20–<22.5, ≥ 22.5–<25.0, ≥ 25.0–<27.5, or ≥ 27.5 kg/m^2^) [25], living status (living with family, alone, missing), socioeconomic status [“easy” or “somewhat easy” (considered equivalent to rich), “somewhat hard” or “hard” (considered equivalent to poor), missing], self-reported health [“very healthy” or “somewhat healthy” (considered equivalent to good), “not very healthy” or “unhealthy” (considered equivalent to poor), missing], self-reported physical fitness [“extremely confident” or “somewhat confident” (considered equivalent to good), “slightly anxious” or “very anxious” = (considered equivalent to poor), missing], sleeping status [“yes” and “somewhat” (considered equivalent to good), “not very much” and “not at all” (equivalent to poor sleep), missing], smoking history (never, “used to, but quit,” i.e., past-smoker, “almost daily” or “sometimes,” i.e., current-smoker, missing), alcohol use (“never” or “almost never,” i.e., non-drinker, “almost daily,” “sometimes,” i.e., drinker, missing), meal frequency (≥ 3, ≤ 2, missing), awareness of the word “frailty” [“know well” or “know a little” or “heard” (equivalent to known), unknown, missing], frailty assessed by the KCL (robust, frailty, missing), and frailty assessed by the FSI (robust, frailty, missing). All the above-mentioned variables were presented as numbers and percentages. Comparisons of the characteristics between the face-to-face and mail surveys were performed using the chi-square test and any missing data were excluded (Tables 1 and 2).

**Table 1:** Characteristics of the participants of the face-to-face and mail surveys

	Face-to-face survey n = 498		Mail survey n = 8815		p value
Age[years]					
<65	106	21.3%	4367	49.5%	<0.001
≥65	392	78.7%	4448	50.5%	
Sex					
Women	412	82.7%	4569	51.8%	<0.001
Men	86	17.3%	4246	48.2%	
Self-reported BMI[kg/m^2^]^a^					
<18.5	40	8.0%	561	6.4%	0.001
18.5–20.5	50	10.0%	981	11.1%	
20–22.5.5	179	35.9%	2614	29.7%	
22.5–25.5	135	27.1%	2369	26.9%	
25–27.5.5	62	12.4%	1307	14.8%	
≥27.5	32	6.4%	983	11.2%	
Living status^b^					
Together	381	76.5%	7611	86.3%	<0.001
Alone	115	23.1%	1071	12.1%	
Socioeconomic status^b^					
Rich	366	73.5%	4895	55.5%	<0.001
Poor	131	26.3%	3837	43.5%	
Self-reported health^b^					
Good	423	84.9%	7268	82.5%	0.181
Poor	75	15.1%	1530	17.4%	
Self-reported physical fitness^b^					
Good	263	52.8%	4813	54.6%	0.403
Poor	235	47.2%	3981	45.2%	
Sleeping status^b^					
Good	396	79.5%	6583	74.7%	0.018
Poor	102	20.5%	2215	25.1%	
Smoking history^b^					
Never	397	79.7%	4560	51.7%	<0.001
Past	83	16.7%	2714	30.8%	
Current	18	3.6%	1532	17.4%	
Alcohol use^b^					
None	319	64.1%	4614	52.3%	<0.001
Use	179	35.9%	4168	47.3%	
Meal frequency[times/day]^b^					
≥3	467	93.8%	7505	85.1%	<0.001
≤2	30	6.0%	1117	12.7%	
Awareness "frailty"^b^					
Known	338	67.9%	2444	27.7%	<0.001
Unknown	157	31.5%	6328	71.8%	
Frailty-KCL^b^					
Robust	413	82.9%	7050	80.0%	0.090
Frailty	84	16.9%	1765	20.0%	
Frailty-FSI^b^					
Robust	445	89.4%	7594	86.1%	0.024
Frailty	51	10.2%	1221	13.9%	

^a^Self-reported body mass index (BMI) calculated using the self-reported height and weight

^b^Participants with missing values: Living status (*n* = 2 in the face-to-face survey and 133 in the mail survey); Socioeconomic status (*n* = 1 and 83); Self-reported health (*n* = 0 and 17); Self-reported physical fitness (*n* = 0 and 21); Sleep status (*n* = 0 and 17); Smoking history (*n* = 0 and 9); Alcohol use (*n* = 0 and 33); Meal frequency (*n* = 1 and 193); Awareness of “frailty” (*n* = 3 and 43); Frailty-KCL (*n* = 1 and 0); Frailty-FSI (*n* = 2 and 0)

**Table 2:** Characteristics of participants stratified by age and BMI in the mail survey

Age	<65 years												≥65 years											
BMI	<18.5		≥18.5–<20.0		≥20.0–<22.5		≥22.5–<25.0		≥25.0–<27.5		≥27.5		<18.5		≥18.5–<20.0		≥20.0–<22.5		≥22.5–<25.0		≥25.0–<27.5		≥27.5	
	n = 267		n = 527		n = 1277		n = 1080		n = 643		n = 573		n = 294		n = 454		n = 1337		n = 1289		n = 664		n = 410	
Age [years]	52.4	7.1	51.7	7.0	52.4	7.2	52.8	7.3	52.8	7.1	51.9	7.0	73.8	6.0	73.3	5.8	73.3	5.7	73.2	5.6	73.4	5.5	72.5	5.5
BMI [kg/m^2^]	17.5	0.8	19.3	0.4	21.3	0.7	23.7	0.7	26.0	0.7	30.4	3.1	17.3	1.1	19.3	0.4	21.3	0.7	23.7	0.7	26.1	0.7	30.3	4.4
Sex																								
Women	222	83.1%	423	80.3%	795	62.3%	489	45.3%	256	39.8%	214	37.3%	189	64.3%	268	59.0%	692	51.8%	520	40.3%	291	43.8%	210	51.2%
Men	45	16.9%	104	19.7%	482	37.7%	591	54.7%	387	60.2%	359	62.7%	105	35.7%	186	41.0%	645	48.2%	769	59.7%	373	56.2%	200	48.8%
Living status^a,b^																								
Together	246	92.1%	471	89.4%	1155	90.4%	955	88.4%	573	89.1%	490	85.5%	239	81.3%	366	80.6%	1111	83.1%	1098	85.2%	568	85.5%	339	82.7%
Alone	19	7.1%	48	9.1%	108	8.5%	109	10.1%	56	8.7%	67	11.7%	52	17.7%	84	18.5%	209	15.6%	170	13.2%	86	13.0%	63	15.4%
Socioeconomic status^a,b^																								
Rich	143	53.6%	303	57.5%	723	56.6%	565	52.3%	314	48.8%	253	44.2%	168	57.1%	253	55.7%	822	61.5%	766	59.4%	367	55.3%	218	53.2%
Poor	123	46.1%	221	41.9%	543	42.5%	509	47.1%	327	50.9%	319	55.7%	119	40.5%	192	42.3%	500	37.4%	510	39.6%	283	42.6%	191	46.6%
Self-reported health^a,b^																								
Good	223	83.5%	457	86.7%	1096	85.8%	907	84.0%	510	79.3%	380	66.3%	221	75.2%	368	81.1%	1142	85.4%	1091	84.6%	548	82.5%	325	79.3%
Poor	44	16.5%	69	13.1%	180	14.1%	173	16.0%	132	20.5%	191	33.3%	73	24.8%	83	18.3%	192	14.4%	195	15.1%	114	17.2%	84	20.5%
Self-reported physical fitness^a,b^																								
Good	133	49.8%	310	58.8%	744	58.3%	590	54.6%	333	51.8%	226	39.4%	129	43.9%	248	54.6%	790	59.1%	759	58.9%	352	53.0%	199	48.5%
Poor	134	50.2%	217	41.2%	530	41.5%	488	45.2%	309	48.1%	346	60.4%	163	55.4%	205	45.2%	544	40.7%	525	40.7%	310	46.7%	210	51.2%
Sleeping status^a,b^																								
Good	184	68.9%	361	68.5%	884	69.2%	754	69.8%	424	65.9%	346	60.4%	233	79.3%	370	81.5%	1083	81.0%	1080	83.8%	542	81.6%	322	78.5%
Poor	82	30.7%	166	31.5%	389	30.5%	323	29.9%	219	34.1%	227	39.6%	60	20.4%	83	18.3%	251	18.8%	207	16.1%	120	18.1%	88	21.5%
Smoking history^a,b^																								
Never	165	61.8%	330	62.6%	681	53.3%	526	48.7%	285	44.3%	255	44.5%	174	59.2%	255	56.2%	721	53.9%	617	47.9%	320	48.2%	231	56.3%
Past	47	17.6%	101	19.2%	330	25.8%	310	28.7%	213	33.1%	175	30.5%	74	25.2%	127	28.0%	436	32.6%	506	39.3%	265	39.9%	130	31.7%
Current	55	20.6%	96	18.2%	266	20.8%	243	22.5%	145	22.6%	143	25.0%	45	15.3%	71	15.6%	179	13.4%	163	12.6%	78	11.7%	48	11.7%
Alcohol use^a,b^																								
None	147	55.1%	283	53.7%	578	45.3%	495	45.8%	314	48.8%	296	51.7%	195	66.3%	286	63.0%	754	56.4%	656	50.9%	373	56.2%	237	57.8%
Use	120	44.9%	244	46.3%	699	54.7%	584	54.1%	328	51.0%	274	47.8%	97	33.0%	166	36.6%	570	42.6%	625	48.5%	288	43.4%	173	42.2%
Meal frequency [times/day]^a,b^																								
≥3	222	83.1%	428	81.2%	1040	81.4%	865	80.1%	518	80.6%	461	80.5%	263	89.5%	405	89.2%	1191	89.1%	1156	89.7%	593	89.3%	363	88.5%
≤2	41	15.4%	88	16.7%	211	16.5%	188	17.4%	113	17.6%	100	17.5%	26	8.8%	37	8.1%	111	8.3%	111	8.6%	57	8.6%	34	8.3%
Awareness "frailty" ^a,b^																								
Known	78	29.2%	158	30.0%	313	24.5%	237	21.9%	137	21.3%	125	21.8%	101	34.4%	164	36.1%	442	33.1%	386	29.9%	187	28.2%	116	28.3%
Unknownn	189	70.8%	369	70.0%	962	75.3%	843	78.1%	504	78.4%	448	78.2%	190	64.6%	284	62.6%	880	65.8%	896	69.5%	472	71.1%	291	71.0%
Frailty-KCL																								
Robust	209	78.3%	459	87.1%	1105	86.5%	901	83.4%	513	79.8%	425	74.2%	189	64.3%	357	78.6%	1063	79.5%	1018	79.0%	523	78.8%	288	70.2%
Frailty	58	21.7%	68	12.9%	172	13.5%	179	16.6%	130	20.2%	148	25.8%	105	35.7%	97	21.4%	274	20.5%	271	21.0%	141	21.2%	122	29.8%
Frailty-SFI																								
Robust	242	90.6%	468	88.8%	1144	89.6%	942	87.2%	532	82.7%	438	76.4%	241	82.0%	398	87.7%	1182	88.4%	1135	88.1%	556	83.7%	316	77.1%
Frailty	25	9.4%	59	11.2%	133	10.4%	138	12.8%	111	17.3%	135	23.6%	53	18.0%	56	12.3%	155	11.6%	154	11.9%	108	16.3%	94	22.9%

^a^Participants with missing values in the age < 65 years group: Living status (*n* = 2 in BMI < 18.5, 8 in BMI ≥ 18.5–<20.0, 14 in BMI ≥ 20.0–<22.5, 16 in BMI ≥ 22.5–<25.0, 14 in BMI ≥ 25.0–<27.5, 16 in BMI ≥ 27.5); Socioeconomic status (*n* = 1, 3, 11, 6, 2, 1,); Self-reported health (*n* = 0, 1, 1, 0, 1, 2); Self-reported physical fitness (*n* = 0, 0, 3, 2, 1, 1); Sleep status (*n* = 1, 0, 4, 3, 0, 0); Smoking history (*n* = 0, 0, 0, 1, 0, 0); Alcohol use(*n* = 0, 0, 0, 1, 1, 3); Meal frequency (*n* = 4, 11, 26, 27, 12, 12); and Awareness of “frailty” (*n* = 0, 0, 2, 0, 2, 0)

^b^Participants with missing values in the age ≥ 65 years group: Living status (*n* = 3 in BMI < 18.5, 4 in BMI ≥ 18.5–<20.0, 17 in BMI ≥ 20.0–<22.5, 21 in BMI ≥ 22.5–<25.0, 10 in BMI ≥ 25.0–<27.5, 8 in BMI ≥ 27.5); Socioeconomic status (*n* = 7, 9, 15, 13, 14, 1); Self-reported health (*n* = 0, 3, 3, 3, 2, 1); Self-reported physical fitness (*n* = 2, 1, 3, 5, 2, 1); Sleep status (*n* = 1, 1, 3, 2, 2, 0); Smoking history (*n* = 1, 1, 1, 3, 1, 1); Alcohol use (*n* = 2, 2, 13, 8, 3, 0); Meal frequency (*n* = 5, 12, 35, 22, 14, 13); Awareness of “frailty” (*n* = 3, 6, 15, 7, 5, 3). *BMI* Body mass index

To evaluate the validity of the self-reported BMI, we performed intraclass correlation coefficient (ICC) class 1 (1,1) and Bland-Altman analyses to compare the systematic errors in height, weight, and BMI between the self-reported and objectively measured data in the face-to-face survey.

Odds ratios (OR) and 95% confidence intervals (95%CI) for frailty categorized by BMI (reference ≥ 22.5–<25.0 kg/m^2^) were calculated using multivariable logistic analysis with age (continuous), sex, and survey area (Settu, Hannan) in Model 1. Model 2 comprised living status, socioeconomic status, self-reported health, self-reported physical fitness, sleeping status, smoking history, alcohol use, meal frequency, and awareness of the concept of “frailty” in addition to Model 1’s variables of as adjustment variables. The adjustment variables were determined based on a previous research [16, 30, 32]. Because an interaction between BMI and age was observed (Table 3), we analyzed the association between BMI and frailty separately for the < 65 and ≥ 65 years age groups.

**Table 3:** Interaction between BMI and age in logistic regression analysis of frailty status

		Model 1	Interaction P-value	Model 2	Interaction P-value
		Odds Ratio (95%CI)	BMI*Age	Odds Ratio (95%CI)	BMI*Age
KCL					
BMI	**<18.5**	0.573 (0.169–1.940.169.940)	0.055	1.075 (0.267–4.323.267.323)	0.436
[kg/m2]	**≥18.5–<20.0**	0.534 (0.177–1.609.177.609)	0.324	1.438 (0.436–4.742.436.742)	0.484
	**≥20.0–<22.5**	0.692 (0.298–1.607.298.607)	0.516	0.992 (0.393–2.507.393.507)	0.967
	**≥22.5–<25.0**	ref	―	ref	―
	**≥25.0–<27.5**	**3.036 (1.175–7.840.175.840)**	**0.042**	**3.615 (1.252–10.44.252.44)**	**0.016**
	**≥27.5**	**2.844 (1.084–7.461.084.461)**	0.326	0.920 (0.311–2.723.311.723)	0.493
Age [years]		**1.022 (1.013–1.031.013.031)**	―	**1.038 (1.028–1.049.028.049)**	―
FSI					
BMI	**<18.5**	**0.096 (0.019–0.476.019.476)**	**0.002**	**0.094 (0.017–0.520.017.520)**	**0.008**
[kg/m2]	**≥18.5–<20.0**	0.519 (0.153–1.764.153.764)	0.304	0.815 (0.228–2.914.228.914)	0.862
	**≥20.0–<22.5**	0.710 (0.275–1.836.275.836)	0.609	0.852 (0.312–2.327.312.327)	0.902
	**≥22.5–<25.0**	ref		ref	―
	**≥25.0–<27.5**	1.581 (0.561–4.454.561.454)	0.903	1.794 (0.587–5.479.587.479)	0.629
	**≥27.5**	1.936 (0.684–5.482.684.482)	0.821	0.833 (0.268–2.591.268.591)	0.219
Age [years]		1.003 (0.992–1.013.992.013)	―	**1.015 (1.003–1.026.003.026)**	―

Model 1: BMI (6 categories), age (continuous)

Model 2: Model 1 + sex, survey area (Settsu or Hannan), living status, socioeconomic status, self-reported health, self-reported physical fitness, sleeping status, smoking history, alcohol use, meal frequency, and awareness of “frailty”

*BMI* Body mass index, *KCL* Kihon Checklist, *FSI* Frailty screening index, *ref* Reference, *95%CI *95% confidence interval

Bold font indicates statistical significance

We also performed a restricted cubic spline analysis using BMI 15 to 35 (0.2 to 99.3 percentiles). Knots of cubic spline analysis were placed the 5th, 50th and 95th percentiles of BMI. We calculated adjusted ORs for frailty associated with BMI using 50th percentiles of BMI as the reference.

Statistical analysis was performed using SPSS 26 (IBM, Armonk, NT, USA), and R software version 4.5.2 (R Development Core Team, Vienna, Austria). Statistical significance was set at *p* < 0.05.

### Ethical approval

The survey plan was approved by the Ethics Review Committee of the National Institute of Health and Nutrition, National Institutes of Biomedical Innovation, Health and Nutrition. The face-to-face survey was approved (Approval No. *I-ki-ken-hatsu 781-1*) and registered with the UMIN Clinical Trials Registry (*UMIN000036880;* Registration date: May 29th 2019; Website: https://upload.umin.ac.jp/cgi-open-bin/ctr_e/ctr_view.cgi?recptno=R000042027). All participants provided written informed consent. Although we submitted an application for the mail survey to the committee (application No. *Ken-ei 89*), it was not reviewed by the committee because only city workers could access personal information, the questionnaires were directly mailed from Settsu city to subjects, and the returned questionnaires were anonymous. The committee considered the mail survey to be a general survey. Article #33 of Japan Statistics Act permits the use general survey data in academic research. The mailed questionnaire included descriptions of the purpose and selection of participants. The envelopes were color-coded by school district. Response to the questionnaire was regarded as consent.

## Results

The participants of the face-to-face survey were older, lived alone, had a higher female preponderance, normal BMI, good economic condition, good sleep status, non-smokers, non-alcohol drinkers, had three and more meals per day, had awareness of the word “frailty,” and had lower rate of frailty, as measured by the KCL and FSI, than the participants of the mail survey (Table 1).

In the face-to-face survey, a high ICC (0.948–0.994) was confirmed for the self-reported and objectively measured height, weight, and BMI; however, there was a fixed error in height and weight, except height for participants aged < 65 years, and a proportional error in BMI in all participants (Supplementary Table 1, Supplementary Fig. 1).

Table 2 demonstrates the characteristics of participants stratified by age and BMI groups in the mail survey. The characteristics of participants were largely similar in both age groups, although people with higher BMI tended to be poor socioeconomic status in < 65 years and to be rich in ≥ 65 years (Table 2). Table 3 demonstrates the interaction between BMI and age (Table 3).

**Fig. 2:**
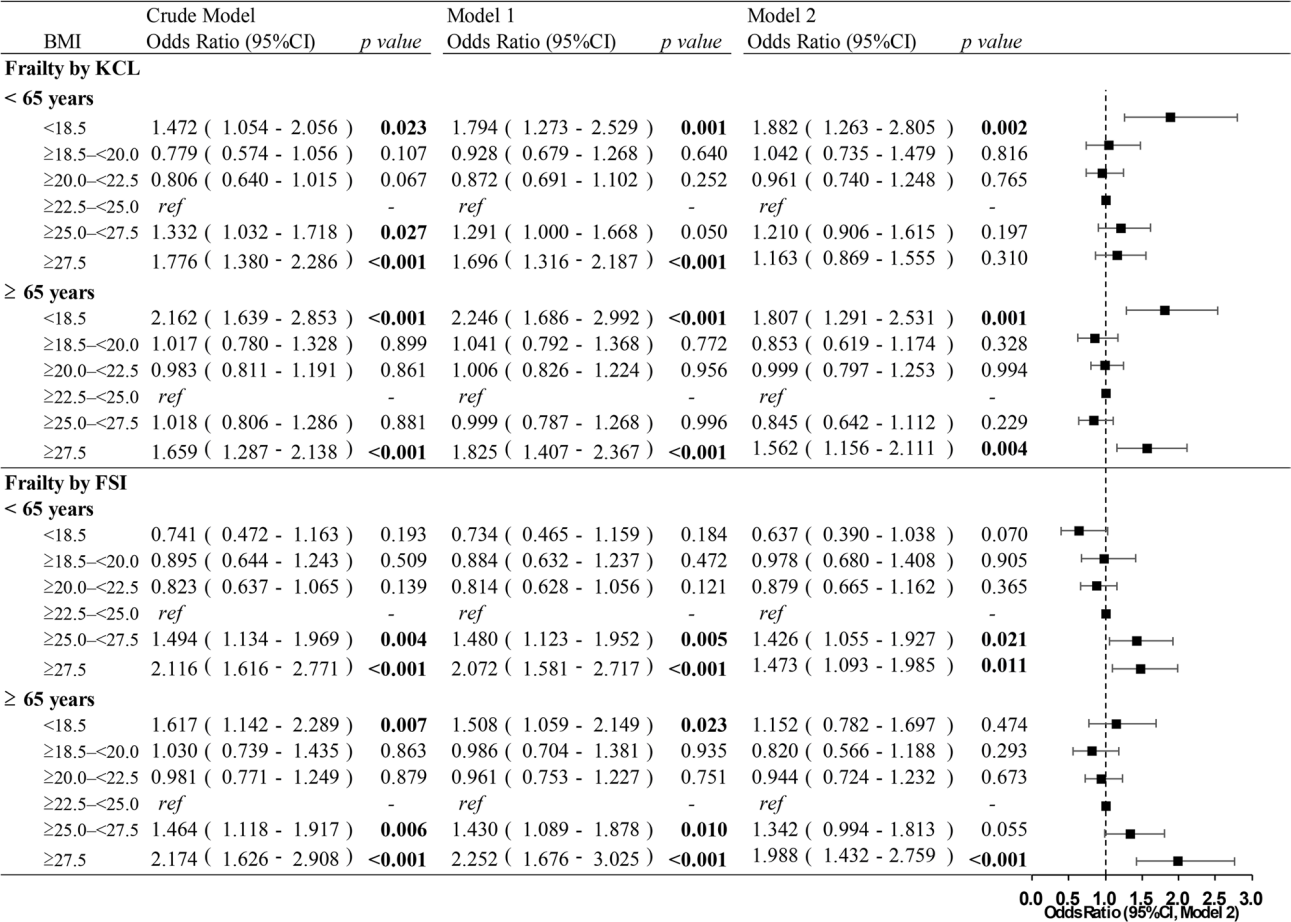
Logistic regression analysis between BMI and frailty status stratified by age. Model 1: Age (continuous), sex, and survey area (Settsu or Hannan). Model 2: Model 1 + living status, socioeconomic status, self-reported health, self-reported physical fitness, sleeping status, smoking history, alcohol use, meal frequency, and awareness of “frailty”. BMI, body mass index; KCL, Kihon Checklist; FSI, Frailty screening index; ref, reference; 95%CI, 95% confidence interval. Bold font indicates statistical significance

Using BMI ≥ 22.5–<25.0 kg/m^2^ as reference, KCL-measured frailty was significantly associated with BMI < 18.5 kg/m^2^, ≥ 25.0–<27.5 kg/m^2^, and ≥ 27.5 kg/m^2^ in the crude model; however, after adjustment in Model 2, only BMI < 18.5 kg/m^2^ (OR = 1.882, 95%CI: 1.263–2.805) retained statistical significance for participants aged < 65 years. For individuals aged ≥ 65 years, BMI < 18.5 kg/m^2^ (OR = 1.807, 95%CI: 1.291–2.531) and ≥ 27.5 kg/m^2^ (OR = 1.562, 95%CI: 1.156–2.111) retained significance even after adjustment (Fig. 2).

Frailty measured by the FSI was significantly associated with BMI ≥ 25.0–<27.5 kg/m^2^ (OR = 1.426, 95%CI: 1.055–1.927) and ≥ 27.5 kg/m^2^ (OR = 1.473, 95%CI: 1.093–1.985) for participants aged < 65 years. For those aged ≥ 65 years, frailty was significantly associated with BMI < 18.5 kg/m^2^, ≥ 25.0–<27.5 kg/m^2^, ≥ 27.5 kg/m^2^ in the crude model; however, only BMI ≥ 27.5 kg/m^2^ (OR = 1.988, 95%CI: 1.432–2.759) retained statistical significance after adjustment in Model 2 (Fig. 2).

**Fig. 3:**
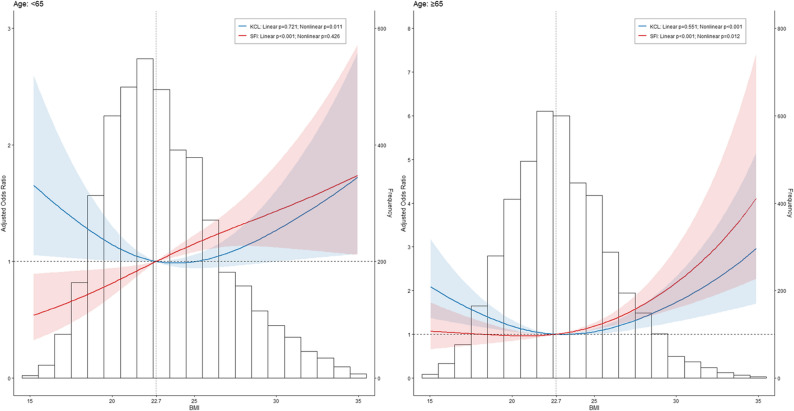
Restricted cubic spline analysis for BMI and frailty status. Model: Age (continuous), sex, survey area (Settsu or Hannan), living status, socioeconomic status, self-reported health, self-reported physical fitness, sleeping status, smoking history, alcohol use, meal frequency, and awareness of “frailty”. OR, odds ratio; BMI, body mass index; KCL, Kihon Checklist; FSI, Frailty screening index. The 50th percentiles of BMI was used as the reference. Blue line and ribbon represent KCL-measured frailty, whereas red line and ribbon represent FSI-measured frailty. Odds ratio and 95% confidence interval are indicated

The association between BMI and frailty using restricted cubic spline models is demonstrated in Fig. 3. Low and high BMIs were significantly associated with frailty for participants aged ≥ 65 years. However, low BMI was not significantly associated with frailty for participants aged < 65 years in FSI (Fig. 3).

## Discussion

To the best of our knowledge, this is the first study to assess the association between BMI and frailty using two frailty tools in middle-aged community-dwelling adults in Japan. Additionally, we confirmed the reproducibility of this association in community-dwelling older adults.

In the crude model, both low and high BMI were associated with frailty measured by the KCL in middle-aged and older adults, and both low and high BMI were associated with frailty measured by the FSI in older adults. Even after adjustment, low and high BMI retained the association with KCL-measured frailty in older adults, showing a U-shaped relationship; however, high BMI lost its significance after adjustment for frailty in middle-aged individuals, showing an L-shaped relationship. The association between low BMI and FSI-measured frailty in older individuals disappeared after adjustment, showing a J-shaped relationship. The association between BMI and frailty in restricted cubic spline models was similar result of the logistic regression analysis. A previous study reported a U-shaped association between BMI and frailty assessed by the KCL and SFI in community-dwelling individuals aged ≥ 65 years in Japan [25]. The results for KCL-measured frailty in older adults in this study were similar to those of previous studies; however the results for FSI-measured frailty in older adults and KCL-measured frailty in middle-aged participants did not fully support or expand upon the results of previous studies. The previous and current studies differed in some aspects, such as the adjustment variables, and frailty assessment tool (the previous study used the modified FSI), which might have affected the detection power. Therefore, the conclusion regarding the J-shaped association between BMI and FSI-measured frailty in older adults and L-shaped relationship BMI and KCL-measured frailty in middle-aged adults was withheld.

The result of the FSI for middle-aged adults across all models consistently showed that only a high BMI was associated with frailty. Although a low BMI did not show a significant relationship with frailty, the odds ratio of frailty was lower. Based on this result, three hypotheses were posited regarding the association between low BMI and frailty in middle-aged individuals: (1) low BMI and frailty are truly associated, but misclassification of frailty occurred in this study, (2) low BMI is not associated with frailty in this study, and (3) low BMI is associated with frailty suppressively.

In middle-aged individuals, low BMI may reduce the risk of lifestyle-related diseases. The association between body size and subjective health differed dynamically depending on age: the group with BMI < 18.5 kg/m^2^ had the highest subjective health compared with other BMI groups in the young age group, but the lowest in older population [37]. A previous study has shown that low subjective health is a moderate predictor for frailty [38], and the current study showed that the association between low BMI and frailty differed between middle-aged and older individuals, supporting the results of the above-mentioned study at first glance. However, low BMI is a marker for decreasing skeletal muscle mass in conditions, such as sarcopenia [47], which is the main component of frailty, and also for malnutrition [39]. The cycle of frailty [18, 33] simulates a negative spiral, including the frailty phenotype, based on the physiological energy cycle and onset of sarcopenia due to malnutrition. Considering this spiral model of frailty, low BMI is a strong factor explaining frailty; however, it is inconsistent with the fact that low BMI was not associated with frailty (assessed by the FSI) in the middle-aged participants in this study. In this study, a low BMI was associated with frailty as assessed by the KCL in middle-aged individuals; therefore, misclassification might occur if only the FSI is used, as it mainly assesses the physical aspects of frailty. As self-reporting is influenced by personal bias, it is necessary to conduct follow-up examinations using objectively measured frailty.

BMI might have different implications for middle-aged and older individuals, such as weight loss rather than being underweight. Being underweight, resulting from weight loss due to certain diseases, has adverse effects on health [37]. In addition, as older adults become shorter in height [40], their BMI increases. For example, if a person weighs 50 kg and their height changes from 150 to 149 cm, the BMI increases by 0.3 kg/m^2^. The assumption that the implications of BMI differ between middle-aged and older individuals might also explain the difference in the association between BMI and frailty between middle-aged and older adults.

Using a statistical model to draw a trajectory of the Frailty Index (FI) score with age, Raymond et al. [41] reported that the FI score increased twice as fast with age = 65 years as a turning point in a 27-year follow-up study in Sweden. Interestingly, this study categorized participants by weight and found that the underweight group had the lowest FI compared to other weight groups up to the age of 60 years; however, after the age of 60 years, the FI increased rapidly and was the highest in the underweight group compared to the other groups. The results of this study were consistent with those of previous studies showing that being underweight suppressed the FI in middle-aged adults but was higher in older adults. As neither possibility can be ruled out, it is difficult to draw definitive conclusions regarding the association between low BMI and frailty as assessed by the FSI in middle-aged individuals.

This study had several limitations. First, the temporal association between BMI and frailty could not be clarified owing to the cross-sectional design. Second, the response rate of both mail surveys was approximately 60%, which might have resulted in selection bias by virtue of including highly health-conscious residents.

Third, the validities of the KCL and FSI were verified in older adults. Using the same dataset, we indirectly showed that the KCL and FSI were partially applicable as the association was the same for older and middle-aged individuals [16]; however, it is unclear whether these tools are appropriate for middle-aged individuals.

Fourth, the exposure factors and outcomes were self-reported. However, performing actual measurements in large-scale surveys is difficult. We confirmed a high ICC between the actual and self-reported height, weight, and BMI in another sample from the same region; therefore, the self-report error was assumed to be small. Blodgett et al. reported that, while one in four people scored 0 on the self-reported FI, less than one in 40 people scored 0 for laboratory-measured FI, suggesting that the onset of asymptomatic frailty occurs before its clinical manifestation [17]. Actual measurements may be more accurate than self-reported measurements in assessing frailty. However, a previous study showed that self-reported frailty assessment can predict independence in the activities of daily living more accurately than epigenetic clocks, such as leukocyte telomere length and DNA methylation [42], and another study showed that self-reported frailty assessment and some types of DNA methylation age were useful for predicting mortality [43]. Insufficient evidence for the accuracy of self-reported and measured frailty was also a limitation of this study.

Fifth, the definition of frailty has no global consensus. Similarly, the definitions of underweight and obesity in BMI vary among populations. In this study, participants with high BMI were few. Future research is needed to confirm the robustness of the findings.

BMI and body weight are widely used clinically because they are easy to measure. However, body composition is more important for health. Recent research has considered calf circumference as an indirect marker of muscle mass including it in the statistical model of the association between BMI and frailty, the results remained unchanged after adjusting for calf circumference [44]. Future research is warranted to explore the association between body composition and frailty, with a focus on research method appropriate for a population-wide study.

## Conclusion and perspective

This study clarified the association between BMI and frailty among middle-aged adults and examined its reproducibility among older adults in Japan. Although the conclusion regarding these associations for middle-aged individuals was withheld, it was implied that both low and high BMI were associated with frailty. The results did not completely replicate those of previous studies in older adults. However, when all the results of this study were collated, we inferred that lifestyle habits that promote proximity to “normal weight” may help prevent frailty. This approach may help solve the double burden of underweight and obesity according to the Sustainable Development Goals target 2.2 [45]; moreover, it might be reasonable to implement measures against frailty in parallel with other weight-related diseases. For example, a “metabolic-and-frail syndrome checkup” for early detection and treatment incorporates some elements of frailty assessment to that of metabolic syndrome, a typical lifestyle-related condition. Monitoring individuals with abnormal BMI could benefit public health [41]. A recent study reported that the association between BMI and mortality risk in older adults varies greatly depending on the presence/absence of frailty [46]. Future detailed studies with better designs are needed to estimate the associations among BMI, frailty, and age.

## Supplementary Information

Supplementary Material 1.

Supplementary Material 2.

## Data Availability

Researchers can request our study group for permission to use this data by contacting T.Y. (tsukasa.yoshida.a3@tohoku.ac.jp).

## Abbreviations

BMI: Body mass index
CI: Confidence interval
FI: Frailty Index
FSI: Frailty screening index
ICC: Intraclass coefficient
KCL: Kihon checklist
OR: Odds ratio
SFI: Simplified Frailty Index
