# Supplementary material for: Association between body mass index and frailty for middle-aged and older adults in Japan: a cross-sectional study of the Osaka health disparity solution program
Source: BMC Public Health. 2026 Apr 23;26:1557. doi: 10.1186/s12889-026-27331-2 (PMC13173948; doi:10.1186/s12889-026-27331-2)
Supplement: Supplementary file 1 — Supplementary Material 1. [file 12889_2026_27331_MOESM1_ESM.docx]

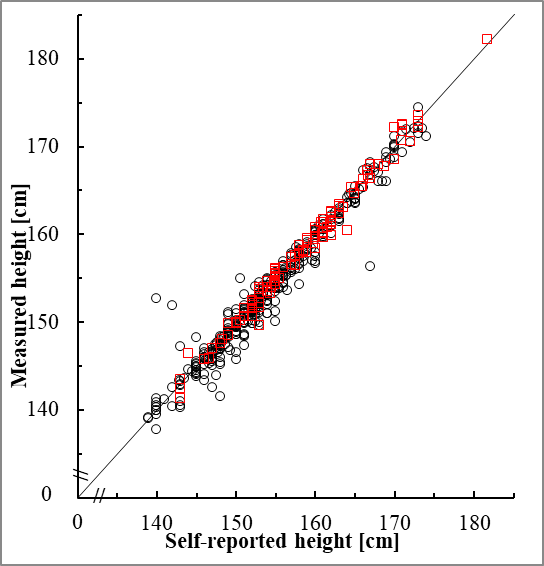

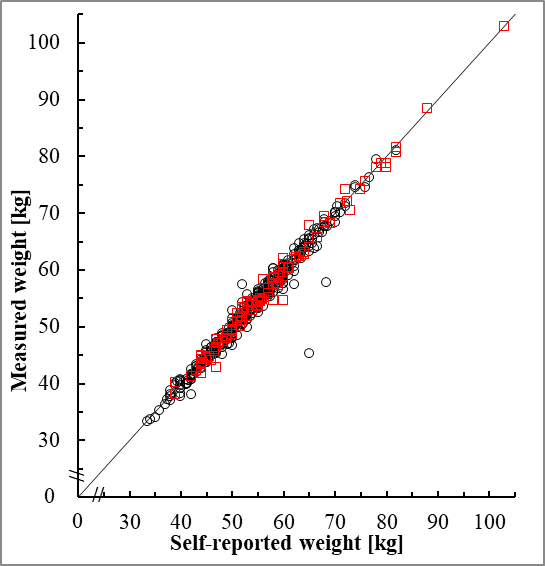


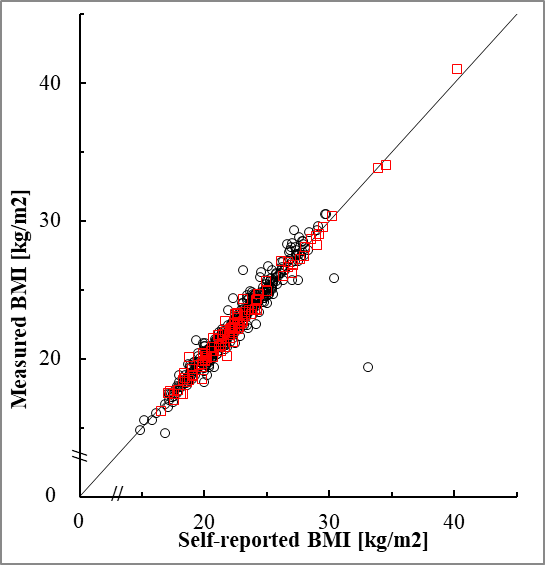


Supplementary Figure 1. Scatter-plots of the intraclass correlation coefficients comparing the self-reported and measured height, weight, and BMI in the age <65 years (red square) and ≥65 years (black circle) groups
